# Sialyltransferase Mutations Alter the Expression of Calcium-Binding Interneurons in Mice Neocortex, Hippocampus and Striatum

**DOI:** 10.3390/ijms242417218

**Published:** 2023-12-07

**Authors:** Senka Blažetić, Vinko Krajina, Irena Labak, Barbara Viljetić, Valentina Pavić, Vedrana Ivić, Marta Balog, Ronald L. Schnaar, Marija Heffer

**Affiliations:** 1 Department of Biology, Josip Juraj Strossmayer University of Osijek, Ulica cara Hadrijana 8A, 31000 Osijek, Croatia; senka@biologija.unios.hr (S.B.); vpavic@biologija.unios.hr (V.P.); 2 Department of Medical Biology, School of Medicine, Josip Juraj Strossmayer University of Osijek, 31000 Osijek, Croatia; krajina.vinko@gmail.com (V.K.); vivic@mefos.hr (V.I.); mbalog@mefos.hr (M.B.); mheffer@mefos.hr (M.H.); 3 Department of Chemistry, Biochemistry and Clinical Chemistry, School of Medicine, Josip Juraj Strossmayer University of Osijek, 31000 Osijek, Croatia; bviljetic@mefos.hr; 4 Department of Pharmacology and Neuroscience, The Johns Hopkins School of Medicine, Baltimore, MD 21205, USA; schnaar@jhu.edu

**Keywords:** *St3gal2*, *St3gal3*, calcium-binding interneurons, cortex, striatum, hippocampus

## Abstract

Gangliosides are major glycans on vertebrate nerve cells, and their metabolic disruption results in congenital disorders with marked cognitive and motor deficits. The sialyltransferase gene *St3gal2* is responsible for terminal sialylation of two prominent brain gangliosides in mammals, GD1a and GT1b. In this study, we analyzed the expression of calcium-binding interneurons in primary sensory (somatic, visual, and auditory) and motor areas of the neocortex, hippocampus, and striatum of *St3gal2*-null mice as well as *St3gal3*-null and *St3gal2/3*-double null. Immunohistochemistry with highly specific primary antibodies for GABA, parvalbumin, calretinin, and calbindin were used for interneuron detection. *St3gal2*-null mice had decreased expression of all three analyzed types of calcium-binding interneurons in all analyzed regions of the neocortex. These results implicate gangliosides GD1a and GT1b in the process of interneuron migration and maturation.

## 1. Introduction

Interneurons are central nervous system nerve cells that integrate sensory (afferent) and motor (efferent) signals in various neural circuits. They most often use gamma-aminobutyric acid (GABA) to inhibit their targets. The functionality of interneurons manifests in the formation of synapses on certain efferent neurons after receiving the synaptic signals from sensory afferent neurons. In most regions of the central nervous system (CNS), families of inhibitory interneurons exist with specific roles in each neural circuit [1]. There are two types of neurons in the cerebral cortex: excitatory pyramidal cells (70–80%) and inhibitory interneurons (20–30%) [2]. The interneurons of the cerebral cortex are cells with short axons, i.e., axons that do not emerge from the neocortex [3]. The body of the interneuron is in the cerebral cortex, with a role in controlling and synchronizing signals coming from pyramidal cells. They are present in all layers of the cerebral cortex, especially in layer IV. The influence of GABAergic interneurons on pyramidal cells depends on the subspecies of interneurons and their location within the cerebral cortex [4]. GABAergic interneurons are a subset of neurons that release GABA as their primary neurotransmitter. GABA is the main inhibitory neurotransmitter in the central nervous system. Interneurons play a critical role in maintaining the balance of excitation and inhibition in neural circuits. They provide inhibitory inputs to other neurons, influencing their firing patterns and overall network activity.

The striatum, the main part of the basal ganglion associated with motor and cognitive functions, is the main target location for afferent neurons of the cerebral cortex and thalamus [5]. In rodents, most striatal neurons are GABAergic spiny projection neurons [6]. Unlike the striatum, in the hippocampus which has an important role in memory, learning, spatial orientation, behavior, and many different disorders [7], GABAergic interneurons present only 10–15% of total neurons [8].

There are different ways of interneuron classification, and one of them is based on neuropeptides synthesized together with GABA. Calcium ions (Ca^2+^) are important secondary messengers. To achieve precision in calcium signaling, cells express specific proteins that ensure the flow of calcium through the cell membrane. As such we call the proteins calcium-binding proteins (CaBP). Among the proteins that can bind calcium, the most common are those belonging to the EF hand family of proteins [9,10].

Based on the presence of calcium-binding proteins, interneurons are divided into parvalbumin (PV) baskets and Chandelier cells that broadcast actions with potential high frequency that are found in layers IV and V of the cortex, calbindin (CB) bipolar and multipolar cells located in layers I, II, and III, and calretinin (CR) bipolar cells located in layers I–IV. PV interneurons participate in signaling between columns, while CB and CR interneurons are involved in signaling within columns [11]. Morphologically distinct cell types express different CaBP, but they also overlap [12]. PV, CB and CR are three CaBP that standout among the numerous CaBPbased on the specificity of their distribution; they are present in distinct morphological classes of inhibitory interneurons [13,14,15].

Overall, the connection between calcium and interneurons is fundamental to the proper functioning of neural circuits. Dysregulation of calcium homeostasis in interneurons has been implicated in various neurological disorders, and understanding the intricacies of calcium signaling in these cells is crucial for unraveling the complexities of brain function [16,17,18,19].

Recognition and interactions between neurons are mediated by glycans [20]. Gangliosides, sialylated glycosphingolipids that are the major glycans of vertebrate nerve cells [21], have great potential to modulate neural calcium [22] and could play fundamental roles in interneuron development and function. There are numerus studies suggesting that gangliosides induce changes in cellular calcium, especially GM1 [23]. Among other things, gangliosides have an important role in neural migration and neurite outgrowth [24], which implicates them in normal physiological and behavioral development and the prevention of neural disorders. The brain is characterized as expressing gangliosides of higher complexity [25], with a dominance of GM1, GD1a, GD1b, and GT1b (97% in adult human brains) [26]. Genetically modified mice lines targeting specific ganglioside biosynthetic genes develop neuronal phenotypes [20], and human congenital disorders of ganglioside biosynthesis are marked by motor and behavioral deficits.

Understanding the interplay between calcium, CaBP, GABA, and interneurons is crucial for unraveling the mechanisms that govern the balance of excitation and inhibition in neural circuits. Dysregulation of these processes can contribute to neurological disorders, emphasizing the importance of studying these interactions in both health and disease.

This study reports the impacts of *St3gal2* and *St3gal3* deficiency, genes responsible for almost all terminal sialylation of brain gangliosides in mice [21]. Furthermore, we study the impact of *St3gal2* and *St3gal3* deficiency on the differentiation and distribution of calcium-binding interneurons (PV, CB, and CR) in different parts of the brain.

The hypothesis in this study is that the ability to biosynthesize gangliosides in developmentally specific relationships participates in directing the differentiation of neurons according to typical chemical phenotypes and that the consequences of a lack of synthesis is seen as changes in the number and types of mature interneurons.

## 2. Results

### 2.1. Ganglioside Distribution in the Brain of Genetically Modified St3gal2-null, St3gal3-null, and St3gal2/3-double null Mouse Models

**Figure 1:**
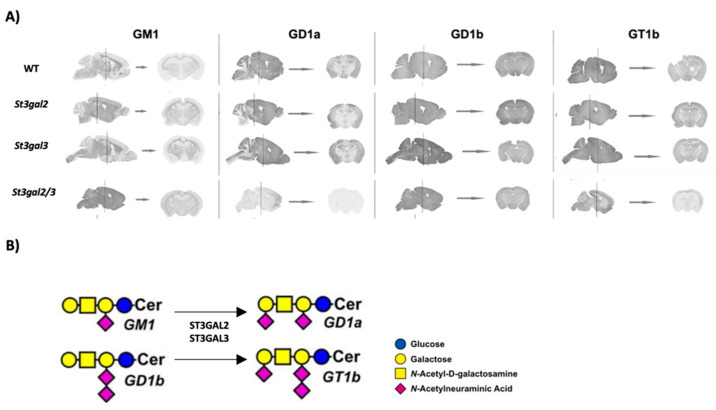
(**A**) The immunohistochemical distribution of major brain gangliosides (GM1, GD1a, GD1b, and GT1b) on sagittal and coronary brain sections of genetically modified mouse models (*St3gal2*-null, *St3gal3*-null, and *St3gal2/3*-double null) and wild-type mice (WT). (**B**) Schematic overview of the structures and biosynthetic pathways of the major brain gangliosides (GM1, GD1a, GT1b and GT1b) with responsible biosynthetic enzymes ST3GAL2 and ST3GAL3.

Genetically modified mouse models *St3gal2*-null, *St3gal3*-null, and *St3gal2*/3-double null have different expression and distribution patterns of major brain gangliosides compared to control mice (WT), as was previously published [27]. *St3gal2* sialyltransferase is responsible for adding the outermost sialic acid to the major brain gangliosides GD1a and GT1b. In its absence, there is a reduction in GD1a and GT1b with coordinate increases in their precursors GM1 and GD1b, respectively (Figure 1B). Disruption of the *St3gal3* sialyltransferase gene has a minor effect on ganglioside synthesis, but residual GD1a/GT1b synthesis in *St3gal2*-null mice is absent in *St3gal2/3*-double null mice.

In the current study, we conducted immunohistochemical analyses, both coronal and sagittal, to delineate the specific distribution patterns of gangliosides within distinct regions of the brain (Figure 1A). Our observations revealed the presence of GM1 gangliosides across all genetically modified mouse models under scrutiny, albeit with notable differences in its distribution throughout the brain.

In wild-type (WT) mice, GM1 gangliosides manifested primarily within myelinated fibers, predominantly in the lower strata of the cerebellum cortex, the nuclei of the thalamus, and the white matter of the cerebellum. Notably, *St3gal3*-null mice exhibited the least deviation from this expression pattern when compared to WT mice, whereas both *St3gal2*- and *St3gal2/3*-double null mice displayed augmented GM1 expression levels. In the latter two strains, GM1 was detectable in all layers of the neocortex and within the CA1 and CA3 regions of the hippocampus. In contrast, neither the neocortex nor the hippocampus of WT and *St3gal3*-null mice exhibited any discernible increase in GM1 (Figure 1A).

Furthermore, the *St3gal2/3*-double null mice demonstrated a substantial elevation in GM1 expression across all analyzed brain regions relative to the WT group. Remarkably, the corpus callosum stood out as the epicenter of the most robust GM1 expression within all the mice under investigation (Figure 1A).

In wild-type (WT) mice, the ganglioside GD1a exhibits a predominant presence in the gray matter of the brain, along with specific neural pathways in the white matter. Notably, GD1a is discernible across multiple brain regions, including all layers of the neocortex, the striatum, the globus pallidus, the amygdala, all strata of the hippocampus and its dentate gyrus, as well as the nuclei of the thalamus and hypothalamus. Furthermore, GD1a is notably expressed in the molecular, granular, and Purkinje layers of cerebellar cells (Figure 1A).

Conversely, in *St3gal2*-null mice, a noticeable reduction in GD1a expression is evident, whereas *St3gal2/3*-double null mice exhibited an almost complete absence of GD1a. In the coronal section, a positive GD1a response is absent due to the presentation of a precaudal incision. These findings indicate a correlation between the abundance of GD1a and regions with lower GM1 expression (Figure 1A).

As for GD1b and GT1b gangliosides, their distribution is largely uniform throughout both the gray and white matter regions of the brain, with one exception in the corpus callosum, which does not exhibit a positive response to GD1b and GT1b. In *St3gal2*-null mice, there is an augmentation in GD1b expression, which is further amplified in *St3gal2/3*-double null mice. Conversely, GT1b expression is reduced in *St3gal2*-null mice, and nearly absent in *St3gal2/3*-double null mice, except for its residual presence solely in the V layer of the cerebral cortex and the CA3 region of the hippocampus (Figure 1A).

### 2.2. Immunohistochemical Expression of Calcium-Binding Interneurons in the Neocortex of Genetically Modified St3gal2-null, St3gal3-null, and St3gal2/3-double null Mouse Models

**Figure 2:**
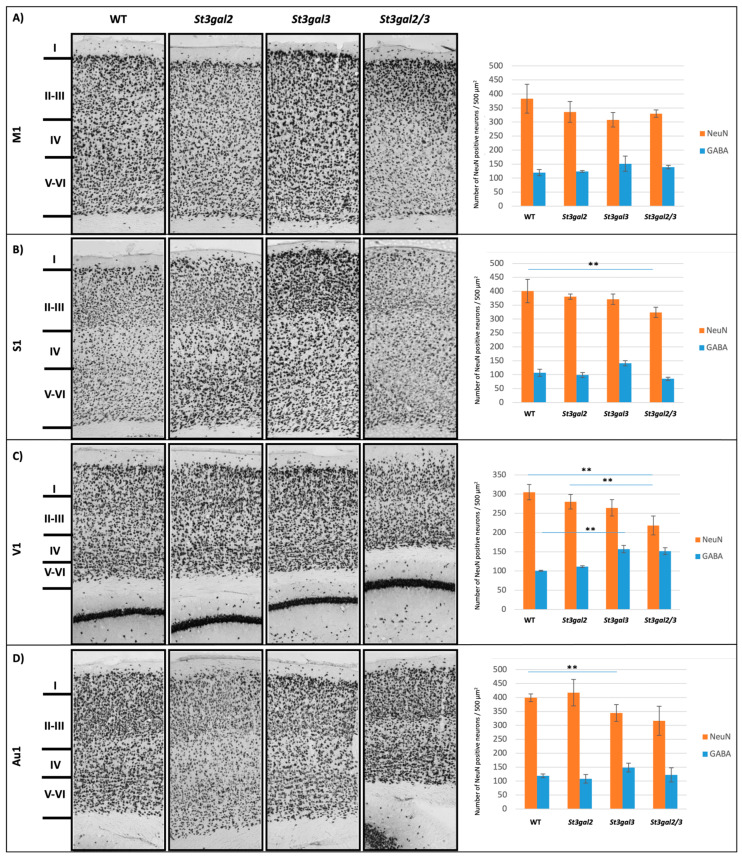
NeuN and GABAergic interneuron expression on coronal brain sections. (**A**) The immunohistochemical expression in the primary motor region (M1) and quantitative analyses (right); (**B**) the immunohistochemical expression in the primary sensory region (S1) and quantitative analyses (right); (**C**) the immunohistochemical expression in the primary visual region (V1) and quantitative analyses (right); (**D**) the immunohistochemical expression in the primary auditive region (Au1) and quantitative analyses (right). Figure also presents layers of the cortex that were used for layer-specific quantification of interneurons. Pictures were taken under 400 magnifications. ** *p* < 0.01.

In the primary motor region (M1) of wild-type (WT) mice, the quantified density of mature neurons expressing the neuronal marker NeuN was found to be 386 ± 51 per measurement field. Notably, the genetically modified mouse models, namely *St3gal2*-null, *St3gal3*-null, and *St3gal2/3*-double null, did not exhibit statistically significant variations in the overall count of NeuN-positive neurons when compared to WT mice (Figure 2A).

Within the primary sensory region (S1) of WT mice, NeuN-positive neurons were observed at a density of 400 ± 42 neurons per measurement field. In *St3gal2/3*-double null mice, a statistically significant reduction in NeuN-positive neuron count was detected (*p* = 0.024) (Figure 2B). Conversely, in the primary visual region (V1), NeuN-positive neurons were quantified at 305 ± 19 per measurement field. A statistically significant discrepancy in the total count of NeuN-positive neurons, as well as the number of GABAergic interneurons, was observed among the genetically modified mouse models. Specifically, in *St3gal2/3*-double null mice, a significant decrease in the total neuron count compared to WT mice was evident (*p* = 0.005). Moreover, a notable difference was observed between *St3gal2*-null and *St3gal2/3*-double null mice (*p* = 0.031). Interestingly, *St3gal3*-null mice exhibited a statistically significant surplus of GABAergic interneurons in comparison to WT mice (*p* = 0.024) (Figure 2C).

In the primary auditory region (Au1), the density of NeuN-positive neurons was determined to be 399 ± 14 per measurement field. Importantly, *St3gal2/3*-double null mice displayed a statistically significant distinction in the overall count of NeuN-positive neurons relative to WT mice (*p* = 0.024) (Figure 2D).

**Figure 3:**
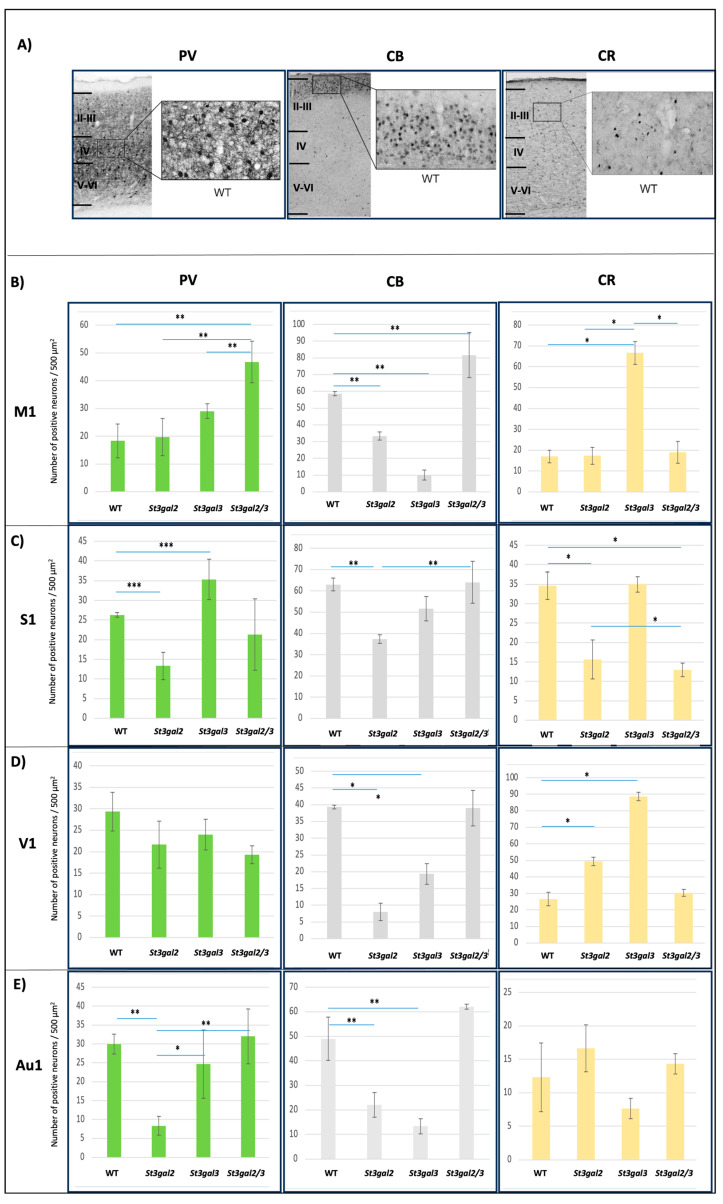
(**A**) The immunohistochemical expression of parvalbumin (PV)-, calbindin (CB)-, and calretinin (CR)-positive interneurons on coronal brain sections in genetically modified mouse models (*St3gal2*-null, *St3gal3*-null, and *St3gal2/3*-double null) and wild-type (WT) mice with marked layers. Pictures were taken under 400 magnifications. (**B**) the number of positive interneurons in the primary motor region (M1); (**C**) the number of positive interneurons in the primary sensory region (S1); (**D**) number of positive interneurons in the primary visual region (V1); (**E**) the number of positive interneurons in the primary auditive region (Au1). * *p* < 0.05, ** *p* < 0.01, and *** *p* < 0.001.

In this study, we observed a consistent positive response in all examined regions of the neocortex in mice concerning the presence of PV, CB, and CR interneurons (Figure 3A).

Notably, the number of PV-expressing interneurons exhibited a significant decrease in the S1 (*p* = 0.007) and Au1 (*p* = 0.010) regions of *St3gal2*-null mice compared to wild-type (WT) mice, with a notable increase in the S1 region of *St3gal3*-null mice (*p* = 0.005). Moreover, the M1 region of *St3gal2/3*-double null mice displayed a significant increase in the number of interneurons expressing PV compared to WT (*p* = 0.002), *St3gal2*-null (*p* = 0.003), and *St3gal3*-null (*p* = 0.029) mice. However, no significant changes were observed in the S1, V1, and Au1 regions (Figure 3B–D).

Conversely, *St3gal2*-null mice demonstrated a reduced number of CB-expressing interneurons across all four neocortical regions, including M1 (*p* = 0.045), S1 (*p* = 0.003), V1 (*p* = 0.023), and Au1 (*p* = 0.001), compared to WT mice. Similarly, *St3gal3*-null mice exhibited fewer CB-expressing interneurons in the M1 (*p* = 0.037), V1 (*p* = 0.046), and Au1 (*p* = 0.005) regions. *St3gal2/3*-double null mice displayed an increased number of CB-expressing interneurons in the M1 region (*p* = 0.015) and a comparable number to WT mice in the S1, V1, and Au1 regions (Figure 3B–D).

Furthermore, the number of CR-expressing interneurons displayed significant changes in different regions among these mutant mice strains. *St3gal3*-null mice showed a significant increase in M1 (*p* = 0.015) and V1 (*p* = 0.021) regions, while the numbers remained unchanged in the S1 and Au1 regions compared to WT mice. Conversely, *St3gal2*-null mice exhibited a contrasting effect on the number of CR interneurons, with a decrease in the S1 region (*p* = 0.035) and an increase in the V1 region (*p* = 0.046). *St3gal2/3*-double null mice only differed from WT mice in the S1 region (*p* = 0.049), where a decrease in the number of CR interneurons was evident. All analyzed regions of the neocortex of all mice showed a positive reaction to the presence of PV, CB, and CR interneurons (Figure 3B–D).

**Figure 4:**
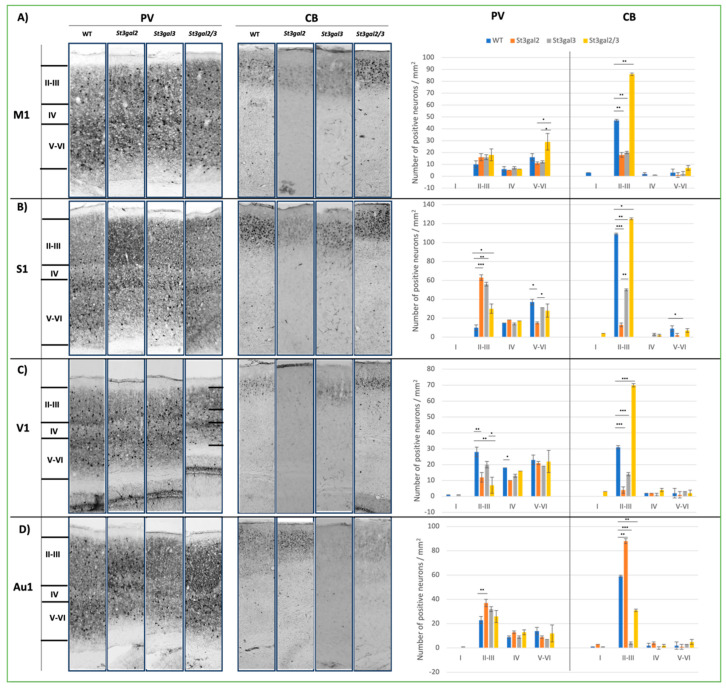
Parvalbumin (PV) and calbindin (CB) interneuron expression through the cortical layers on coronal brain sections in genetically modified mouse models (*St3gal2*-null, *St3gal3*-null, and *St3gal2/3*-double null) and wild-type (WT) mice; (**A**) the immunohistochemical expression in the primary motor region (M1) and quantitative analyses (right); (**B**) the immunohistochemical expression in the primary sensory region (S1) and quantitative analyses (right); (**C**) the immunohistochemical expression in the primary visual region (V1) and quantitative analyses (right); (**D**) the immunohistochemical expression in the primary auditive region (Au1) and quantitative analyses (right). The figure also presents the layers of the cortex that were used for layer-specific quantification of interneurons. Pictures were taken under 400 magnifications. * *p* < 0.05, ** *p* < 0.01, and *** *p* < 0.001.

Moreover, an examination of interneuron distribution across the cortical layers revealed that mutations in sialyltransferase impact the layer-specific expression of CB and PV interneurons. *St3gal2*-null mice exhibited elevated expression of PV neurons in layers 2/3 of the S1 (*p* = 0.013) and Au1 (*p* = 0.022) regions, accompanied by a reduction in the V1 region (*p* = 0.035) (Figure 4B–D). Conversely, CB neurons displayed predominant expression in layers 2/3 across all mice, with a near-complete absence in the other cortical layers. *St3gal2*-null and *St3gal3*-null mice exhibited a significant decrease in the number of CB-positive neurons in the M1 (*p* = 0.012), S1 (*p* = 0.03), and V1 regions (*p* = 0.011). In contrast, in the same regions, *St3gal2/3*-double null mice demonstrated a noteworthy increase in CB-positive neurons (Figure 4A–C).

### 2.3. Immunohistochemical Expression of Calcium-Binding Interneurons in the Hippocampus of Genetically Modified St3gal2-null, St3gal3-null, and St3gal2/3-double null Mouse Models

**Figure 5:**
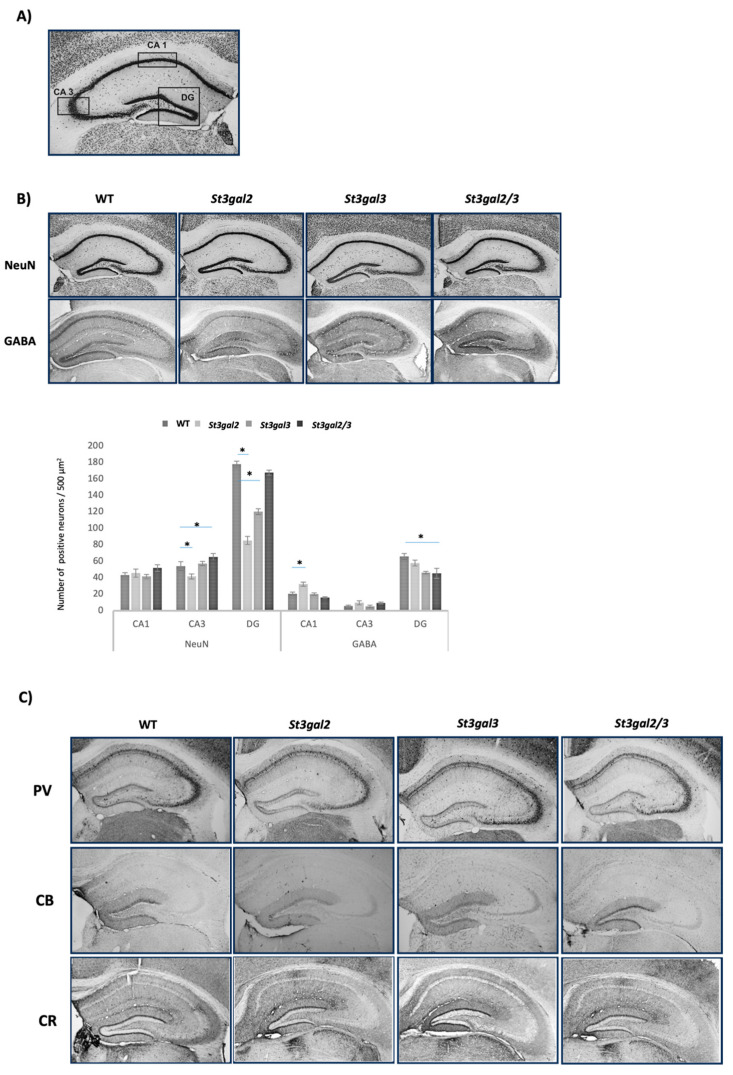
(**A**) Analyzed hippocampal regions, cornu ammonis, CA1, CA2 and dentate gyrus (DG); (**B**) the immunohistochemical NeuN and GABAergic interneuron expression in the hippocampus with quantitative analyses; (**C**) the expression of parvalbumin (PV)-, calbindin (CB)-, and calretinin (CR)-positive interneurons in the hippocampus of genetically modified mouse models (*St3gal2*-null, *St3gal3*-null, and *St3gal2/3*-double null) and wild-type (WT) mice. Pictures were taken under 100 magnifications. * *p* < 0.05.

Hippocampal interneurons were systematically analyzed across three distinct regions: CA1, CA3, and the dentate gyrus (DG) (Figure 5A). Notably, *St3gal2*-null mice exhibited a significant reduction in the total count of neurons expressing the NeuN marker in the CA1 (*p* = 0.010) and DG regions (*p* = 0.02) compared to wild-type (WT) mice. In contrast, *St3gal2/3*-double null mice demonstrated an increase in the CA3 region (*p* = 0.016) relative to WT mice. Furthermore, in comparison to WT mice, the number of GABAergic neurons exhibited distinct patterns of change. Specifically, *St3gal2*-null mice displayed an increase in the number of GABAergic neurons in the CA1 region (*p* = 0.040), while a decrease in the DG region was observed in *St3gal2/3*-double null mice (*p* = 0.030) (Figure 5B).

In all three scrutinized hippocampal regions of wild-type (WT), *St3gal3*-null, and *St3gal2/3*-double null mice, the interneurons exhibited a positive immunoreactivity to PV (Figure 5C). Nevertheless, statistical analysis revealed no significant differences among these strains in terms of PV interneuron expression.

Conversely, CB interneurons were primarily observed in the dentate gyrus (DG) region but did not display any statistically significant variation across the different mouse strains (Figure 5C).

CR interneurons predominantly manifested within the DG region, with a limited presence in isolated cells within the CA1 region. Notably, the CA3 region did not exhibit any discernible immunoreactivity to CR interneurons (Figure 5C).

### 2.4. Immunohistochemical Expression of Calcium-Binding Interneurons in the Striatum of Genetically Modified St3gal2-null, St3gal3-null, and St3gal2/3-double null Mouse Models

**Figure 6:**
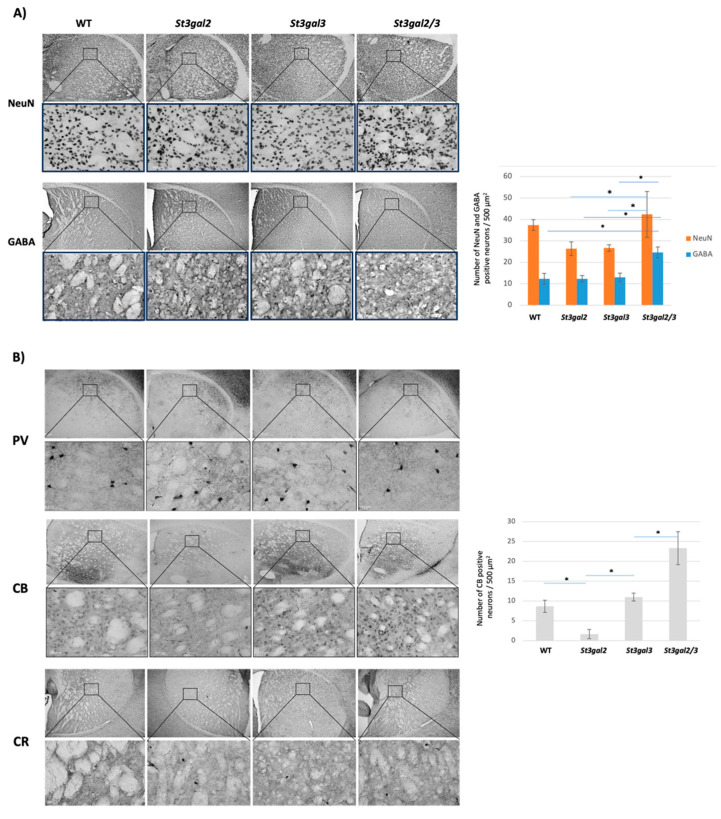
(**A**) The immunohistochemical NeuN and GABAergic interneuron expression in the striatum with quantitative analyses. Pictures were taken under 100 (top row) and 400 (bottom row) magnifications. (**B**) the expression of parvalbumin (PV)-, calbindin (CB)-, and calretinin (CR)-positive interneurons in the striatum in genetically modified mouse models (*St3gal2*-null, *St3gal3*-null, and *St3gal2/3*-double null) and wild-type (WT) mice. Pictures were taken under 400 magnifications. Quantification is only presented for CB because PV and CR did not have any difference. * *p* < 0.05.

The total neuronal count in *St3gal2/3*-double null mice exhibited statistically significant distinctions when compared to *St3gal2*-null (*p* = 0.022) and *St3gal3*-null mice (*p* = 0.042). Immunohistochemical analysis revealed the presence of GABAergic interneurons within the striatum across all mouse models and increased expression in *St3gal2/3*-double null mice compared to WT (*p* = 0.017), *St3gal2*-null (*p* = 0.033), and *St3gal3*-null mice (*p* = 0.01) (Figure 6A).

In this study, a consistent and positive response was observed in the striatum of mice concerning the presence of PV, CB, and CR interneurons. Notably, the only significant difference was detected in the expression of CB interneurons. Specifically, in comparison to wild-type (WT) mice, only *St3gal2*-null mice exhibited a decrease in the count of CB interneurons (*p* = 0.027). Conversely, *St3gal3*-null mice displayed an increase in CB interneurons compared to *St3gal2-null* (*p* = 0.005), while *St3gal2/3*-double null mice exhibited an even greater increase relative to *St3gal3*-null mice (*p* = 0.018) (Figure 6B).

## 3. Discussion

In this study we analyzed interneuron expression in the brains of adult mice after behavioral tests were conducted and resultant specific neural phenotypes were observed [20]. Based on this, we wanted to see if the difference in interneuron expression could possibly impact the neural phenotype. The primary focus of this study was to understand the effect of complex brain gangliosides on mature calcium-binding interneuron expression. *St3gal2*-null mice had reduced expression of GD1a and GT1b, while *St3gal3*-null mice exhibited an almost complete absence GD1a and GT1b. GD1a and GT1b are two of the four major gangliosides in the adult brain, and there are differences in the expression of gangliosides between the young and adult brain; therefore, adult mice brains were used to ensure that the amount of complex brain gangliosides was constant.

This research is the first evidence of a direct connection between the synthesis of complex gangliosides and the distribution of GABAergic interneurons in the brain of genetically modified mice. The presented results refer to a special group of interneurons that express CaBP and exhibit the parvalbumin, calbindin and calreticulin phenotypes. In order to clarify the functions of gangliosides, various genetically altered mouse models with deficient or excessive synthesis of gangliosides or altered genes for enzymes responsible for the biosynthesis or degradation of gangliosides have been developed [28]. From a pathophysiological point of view, disorders of calcium ion homeostasis in the neurons are involved in degenerative diseases, aging, and excitotoxicity, as well as ischemia and hypoglycemia. Changes in the amount and composition of gangliosides have been detected in neurodegenerative diseases such as Alzheimer’s, Parkinson’s, and Huntington’s diseases [29]. Lack of interneurons is a possible link between cognitive dysfunction and variable neuronal activity in various neurodegenerative diseases [30]. It is known that the lack of interneurons is a possible link between cognitive dysfunction and changes in neuronal activity in Alzheimer’s disease [31]. Schizophrenia, autism, and intellectual disabilities represent a spectrum of diseases that have a wide group of possible causes, so a disorder in the structure and function of inhibitory circuits can be one of them. Recent animal and human studies show that the molecular basis of such disorders is associated with certain defects in the development and functioning of interneurons [32,33,34]. Gangliosides are present in different types of neurons: projection (efferent and afferent), association, commissural, and interneurons [15]. It has been proven that we can distinguish between different subtypes of inhibitory interneurons based on the presence of three important proteins that serve as intracellular calcium buffers: parvalbumin, calbindin and calretinin [3]. Considering all the above and the great importance of gangliosides, we analyzed the potential role of gangliosides in the expression of calcium-binding interneurons (PV, CB and CR) in the basic brain regions of genetically altered mouse models: the neocortex, four primary region of the cortex (motor, sensory, auditory, and visual), the striatum, and the hippocampus.

The neocortex is composed of six layers in which neurons are arranged. Most of the neurons of the neocortex are exclusively pyramidal (80%), while the rest are interneurons (20%) [35]. Inhibitory interneurons are of crucial importance for the function of the cerebral cortex and behavior. The mechanisms that control the diversity and distribution of inhibitory neurons in different regions of the cortex are still poorly understood. Generally, *St3gal2*-null mice had reduced expression on PV, CB and CR interneurons in almost all neocortical regions compared to WT mice, while *St3gal3*-null mice and *St3gal2/3*-double null mice had just a few changes that were basically related to an increase in PV and CR interneurons. Additionally, layer-specific expression of CB and PV interneurons of *St3gal2*-null, *St3gal3*-null and *St3gal2/3*-double null mice indicates the importance of GD1a and GD1b in the overall processing and integration of sensory, motor, and associative information.

Those results could be related to fact that ST3Gal-II is primary responsible for terminal sialyation of gangliosides, because *St3gal2*-null mice had significantly reduced GD1a and GT1b [21]. *St3gal2/3*-double null mice had a significant increase in PV interneurons in the M1 region compared to WT mice. Previous research has shown that sialoglycoprotein sialylation is partially blocked in *St3gal3*-null and *St3gal2/3*-double-null mice, but not in *St3gal2*-null mice [27]. The mentioned phenomenon correlates with the related activities of the different regions of the neocortex and probably with the appearance of inhibitory activity of the neocortex [35]. It is known that in rats suffering from Parkinson’s disease there is also an increase in PV-positive interneurons in the M1 region [36], which may be related to the impaired motor activity of *St3gal2/3*-double null mice. Decreased motor activities are a possible consequence of increased pallidal inhibitory activity, which results in akinesia and muscle rigidity [37], while a reduction in GABA inhibition facilitates long-term potentiation in the motor cortex [38,39]. GD1a and GT1b could also interfere with expression of interneurons expressing vasoactive intestinal polypeptide (VIP) because almost all of CB interneurons (214/222) displayed close appositions with VIP boutons on their soma as well [40,41,42].

Increased expression of GM1 gangliosides in *St3gal2/3*-double null mice could also be a factor affecting changes in neurological function, and it is additionally necessary to determine on which interneurons GM1 is expressed. This is supported by a study in which it was confirmed that the injection of GM1 gangliosides into the motor and sensory regions of the rat cortex caused reversible epileptic seizures [43]. The primary motor cortex controls voluntary body movements, while motor-associative areas help in planning and executing motor activity [44]. Interneurons enable the selective and time-coordinated action of sensory and descending motor signals on spinal reflex circuits. Inhibitory interneurons enable the coordinated action of muscles around one joint, and coordinate the action of opposing muscles [45]. *St3gal2*-null mice had decreased expression of all three analyzed types of calcium-binding interneurons, while *St3gal2/3*-double null mice had decreased CR expression. The main role of neurons in the S1 region is to conduct action potentials from receptive sensory areas to the central associative areas of the CNS where sensations are generated [46]. Several studies have shown that the loss of GABAergic interneurons enhances the sensation of pain [47,48,49]. This fact suggests a possible reduced sensitivity to pain in *St3gal3*-null mice. In the V1 region *St3gal2*-null and *St3gal3*-null mice had decreased expression of CR interneurons. CR-positive interneurons form a network that stops the inhibition of pyramidal neurons [50]. Research on the anatomical connections of CR interneurons has revealed variability depending on the study model. In broad terms, the majority of these studies indicate that CR interneurons primarily form connections with the dendrites of other GABAergic cells within the visual cortex of rats [51]. CR interneurons in mice have an overall inhibitory role on cortical activity [52]. In relation to the visual cortex, previous studies using the depth perception test showed that there is no difference between *St3gal2*-null, *St3gal3*-null and *St3gal2/3*-double null mice compared to WT mice [27].

*St3gal2*-null mice had reduced expression of PV and CB interneuron in the Au1 region. Reduced expression of PV interneurons is present in patients with schizophrenia [33,53,54,55] and age-related hearing loss [56]. Additionally, PV-positive interneurons play a role in the initiation of the critical period of cortical plasticity in the visual, sensory, and auditory cortex [57,58,59], and bilateral ablation of the cochlea causes a reduction in GABAergic PV and CB interneurons [60]. All these findings indicate that *St3gal2*-null and *St3gal3*-null mice could possibly disturb sound perception. To confirm this, it is necessary to conduct auditory behavioral tests. In addition, this theory is supported by the fact that in *St3gal*-null and *St3gal3*-null mice there is an increased expression of CR-positive interneurons. Increased expression of CR-positive interneurons occurs after unilateral removal of the Au1 region in mice [61]. Mice only expressing GM3 have been shown to suffer from fatal audiogenic seizures, which may be a consequence of long-term exposure to high-frequency sounds [62]. A general increase in the number of GABAergic neurons in *St3gal2*-null mice in the CA1 region compared to WT mice indicates that GD1a and GT1b gangliosides could have functions in shaping the physiological patterns of the CA1 network.

In conclusion, results from this study point to the possible important role of gangliosides GD1a and GT1b in the process of interneuron migration and maturation. These results support the theory that gangliosides serve as modulators of the availability of divalent cations, and calcium- and magnesium-dependent adhesion systems, thereby influencing ligand binding.

## 4. Materials and Methods

### 4.1. Animal Models

The brains from three genetically modified mice lines with deficiencies in the individual genes *St3gal2*-null and *St3gal3*-null, and a double deficiency of *St3gal2/3*-double null resulting in insufficient synthesis of gangliosides were used in this research [21]. A wild-type (WT) mice model was used as a control because they express all major brain gangliosides (GM1, GD1a, GD1b, and GT1b).

### 4.2. Sample Preparation

The animals were anesthetized with isoflurane (Forane, Baxter Healthcare Corporation, Deerfield, IL, USA), after which transcranial perfusion was carried out. After perfusion, dissection of the entire brain was performed, with samples stored in 4% PFA for the next 24 h. After fixation was completed, the brains were transferred to a 30% solution of sucrose for 24 h. The cryoprotected brains were quickly frozen in isopentane and stored at −80 °C until further analysis.

Brain sections, 35 μm thick, were made in coronary and sagittal directions on a cryostat (Leica, CM3050S, Wetzlar, Germany) at −18 °C. Since free-floating immunohistochemistry was performed, the sections were collected into 1xPBS solution in polystyrene plates with 24 wells (Costar 24-well Plates, Sigma-Aldrich, St. Louis, MO, USA). The sections collected in this way were kept at 4 °C for several days, while sections that were kept for a longer period were transferred to DeOlmos solution and stored at −20 °C.

### 4.3. Immunohistochemistry

**Figure 7:**
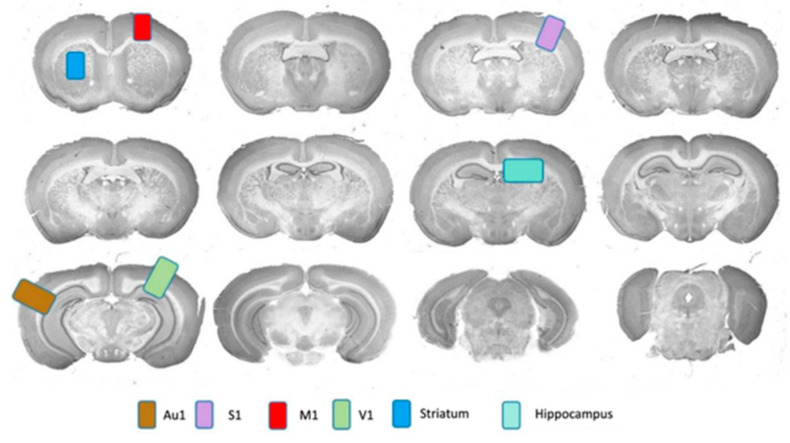
Schematic presentation of the analyzed brain regions according to Bergmann.

Immunohistochemical analysis was performed on the brains of 12 animals (6–12 weeks of age)—3 animals from each mice strain. Each of the genetically modified mice (*St3gal2*-null, *St3gal3*-null, and *St3gal2/3*-double null) as well as WT mice were analyzed in triplicate (three sections from each brain). Four primary regions of the cortex (motor (M1), sensory (S1), visual (V1), and auditive (Au1)), the striatum, and the hippocampus, were analyzed (Figure 7).

Immunohistochemistry was performed based on the modified protocol using highly specific IgG primary monoclonal antibodies to detect gangliosides of all neurons and interneurons [63]. The list and characteristics of all primary antibodies are shown in Table 1. After pretreatment in 0.2% H_2_O_2_ and blocking in (1% BSA, 5% goat serum), sections were incubated overnight at 4 °C in the primary antibodies prepared in a blocking solution in various dilutions as previously determined. Anti-GM1 (1:1000); anti-GD1a (1:2000); anti-GD1b (1:2000); anti-GT1b (1:10,000), anti-Neu (1:2000); anti-GABA (1:1000); anti-PV (1:500); anti-CB (1:200); and anti-CR (1:50). Goat anti-mouse IgG (Jackson Immunoresearch Lab., West Grove, PA, USA, 1:500) was used as a secondary antibody and “Vectastain ABC Kit Elite” (Vector Laboratories, Burlingame, CA, USA) as a tertiary antibody. The DAB Peroxidase Substrate Kit, (Vector Lab, Burlingame, CA, USA) was used for visualization. Control sections were run alongside by the omission of the primary antibodies. Sections were mounted and covered with VectaMount (Vector Laboratories, Newark, CA, USA), scanned in a Super Coolscan 9000 scanner (Nikon, Tokyo, Japan), and analyzed on a microscope (Carl Zeiss, Axioskop 2 MOT, Oberkochen, Germany).

To determine the proportion of GABAergic interneurons in the total number of neurons, an additional analysis of all neocortex neurons using NeuN antibodies was made. After quantitative analysis of the neocortex regions (M1, S1, V1, and Au1), it was determined whether there was a difference in the distribution of NeuN-positive neurons and GABAergic interneurons. Additionally, the difference in the number of PV-, CB-, and CR-positive interneurons in a particular region of the neocortex was analyzed. Quantitative analysis was conducted by counting the entire column with a width of 500 μm, i.e., layer-specific expression based on NeuN staining (Figure 2).

### 4.4. Statistical Analysis

Statistical analysis of the obtained data was made using one-way analysis of variance (ANOVA) for normal data distribution and the Kruskal–Wallis test for unfair distribution. The significance level was 0.05. The analysis was made in the IBM SPSS Statistics V 21.0 program.

## 5. Conclusions

In this paper, for the first time, an analysis of interneurons expressing calcium-binding proteins (parvalbumin, calbindin and calretinin) in the brain of genetically modified *St3gal2*-null, *St3gal3*-null and *St3gal2/3*-double null mouse models was analyzed.

Major findings this study are as follows:

1. The consequence of a lack of ganglioside synthesis is visible as a quantitative change in the different types of mature interneurons.
2. The difference in the number of mature interneurons could be related to the phenotype of individual mice.
3. Sialyltransferases affect the total number of neurons and GABAergic-positive interneurons in the neocortex, of *St3gal2*-null, *St3gal3*-null and *St3gal2/3*-double null mice.
4. In the striatum of all the genetically modified mouse models, there is an increase in the proportion of GABAergic interneurons.
5. In the hippocampus, there is an increase in the expression of CR-positive interneurons in the DG region in *St3gal2*-null, *St3gal3*-null and *St3gal2/3*-double null mice and a decreased in the expression of CB-positive interneurons in *St3gal2*-null and *St3gal3*-null mice.
6. Changes in the expression of PV-, CB- and CR-positive interneurons has a potential role in the development of neurodegenerative disorders.

## Figures and Tables

**Table 1:** The primary antibodies used in this study.

Antigen	Antibody
GM1-1	IgG anti-ganglioside monoclonal antibodies produced and donated by the Department of Pharmacology, The Johns Hopkins School of Medicine (Baltimore, MD, USA) [24]
GD1a-1	
GD1b-1	
GT1b-1	
NeuN	IgG anti-NeuN mouse monoclonal; Abcam (Cambridge, UK), (ab104224)
GABA	IgG anti-GABA mouse monoclonal; Abcam (Cambridge, UK), (ab86186)
Parvalbumin	Unconjugated anti-parvalbumin mouse monoclonal; Abcam, (Cambridge, UK), (ab277625)
Calbindin	Unconjugated anti-calbindin mouse monoclonal; Abcam, (Cambridge, UK), (ab82812)
Calretinin	Unconjugated anti-calretinin mouse monoclonal; Abcam (Cambridge, UK), (ab204990)

## Data Availability

Data is contained within the article.
